# RNA-seq reveals tight junction-relevant erythropoietic fate induced by OCT4 in human hair follicle mesenchymal stem cells

**DOI:** 10.1186/s13287-020-01976-1

**Published:** 2020-10-27

**Authors:** Xiaozhen Yu, Pengpeng Sun, Xingang Huang, Hua Chen, Weiqing Huang, Yingchun Ruan, Weina Jiang, Xiaohua Tan, Zhijing Liu

**Affiliations:** 1 Department of Pathology, College of Basic Medical Sciences, Qingdao University, 308 Ningxia Road, Qingdao, 266071 Shandong China; 2 Department of Critical Care Medicine, Qingdao Center Hospital, affiliated with Qingdao University, 127 Siliunan Road, Qingdao, 266042 Shandong China; 3 Department of Pathology, Qingdao Municipal Hospital, affiliated with Qingdao University, 1 Jiaozhou Road, Qingdao, 266011 Shandong China

**Keywords:** Tight junction pathway, Human hair follicle mesenchymal stem cells, OCT4, Hematopoiesis

## Abstract

**Background:**

Human hair follicle mesenchymal stem cells (hHFMSCs) isolated from hair follicles possess multilineage differentiation potential. OCT4 is a gene critically associated with pluripotency properties. The cell morphology and adhesion of hHFMSCs significantly changed after transduction of OCT4 and two subpopulations emerged, including adherent cells and floating cell. Floating cells cultured in hematopoietic induction medium and stimulated with erythropoetic growth factors could transdifferentiate into mature erythrocytes, whereas adherent cells formed negligible hematopoietic colonies. The aim of this study was to reveal the role of cell morphology and adhesion on erythropoiesis induced by OCT4 in hHFMSCs and to characterize the molecular mechanisms involved.

**Methods:**

Floating cell was separated from adherent cell by centrifugation of the upper medium during cell culture. Cell size was observed through flow cytometry and cell adhesion was tested by disassociation and adhesion assays. RNA sequencing was performed to detect genome-wide transcriptomes and identify differentially expressed genes. GO enrichment analysis and KEGG pathway analysis were performed to analysis the functions and pathways enriched by differentially expressed genes. The expression of tight junction core members was verified by qPCR and Western blot. A regulatory network was constructed to figure out the relationship between cell adhesin, cytoskeleton, pluripotency, and hematopoiesis.

**Results:**

The overexpression of OCT4 influenced the morphology and adhesion of hHFMSCs. Transcripts in floating cells and adherent cells are quite different. Data analysis showed that upregulated genes in floating cells were mainly related to pluripotency, germ layer development (including hematopoiesis lineage development), and downregulated genes were mainly related to cell adhesion, cell junctions, and the cytoskeleton. Most molecules of the tight junction (TJ) pathway were downregulated and molecular homeostasis of the TJ was disturbed, as CLDNs were disrupted, and JAMs and TJPs were upregulated. The dynamic expression of cell adhesion-related gene E-cadherin and cytoskeleton-related gene ACTN2 might cause different morphology and adhesion. Finally, a regulatory network centered to OCT4 was constructed, which elucidated that he TJ pathway critically bridges pluripotency and hematopoiesis in a TJP1-dependent way.

**Conclusions:**

Regulations of cell morphology and adhesion via the TJ pathway conducted by OCT4 might modulate hematopoiesis in hHFMSCs, thus developing potential mechanism of erythropoiesis in vitro.

## Introduction

Erythropoiesis is a stepwise process through which red blood cells (RBCs, erythrocytes) are generated from hematopoietic stem and progenitor cells (HSPCs) and is controlled by multiple elements [1, 2]. Inducing erythrocyte production in vitro provides a model system for exploring the mechanisms of erythropoiesis. Adult mesenchymal stem cells (MSCs) emerged as a promising resource for clinical therapy. Many types of MSCs, such as bone marrow- and adipose-derived mesenchymal stem cells, however, are isolated through invasive operations [3, 4]. Human hair follicle mesenchymal stem cells (hHFMSCs) are a type of MSCs derived from the bulge and papilla of hair follicles, possessing the potential of osteogenic, chondrogenic, and adipogenic differentiation [5]. hHFMSCs are easily available and nonimmunogenic, making them a potential alternative stem cell source for patient-specific applications. OCT4 (POU5F1), the core reprogramming factor, plays an important role in the maintenance of self-renewal and pluripotency of embryonic stem cells (ESCs). Therefore, the researchers transduced single factor OCT4 to hHFMSCs and stimulated with a series of hematopoietic cytokine stimulation to induce OCT4-reprogrammed human hair follicle mesenchymal stem cells (hHFMSCs^OCT4^) transdifferentiate towards the hematopoietic lineage [6]. After transduction, a population of small round floating cells with subtle expression of hematopoietic stem cell (HSC) marker CD45, gradually emerged from hHFMSCs^OCT4^, and could transdifferentiate into mature enucleated RBCs when stimulated with a combination of hematopoietic cytokines [6], while the remaining cells formed negligible hematopoietic colony. This prompted us to consider an association between this particular cell morphology and adhesion with possible erythropoiesis mechanisms, that is, low adhesion and round-like cell morphology conferring higher hematopoietic capacity to hHFMSCs^OCT4^ when treated with cytokines, thus promoting transduction of cellular signals and subsequently initiating the process of erythropoiesis.

It is well known that blood cells grow in suspension, and the process of erythropoiesis is accompanied by great changes in cell morphology. The cell size gradually increases as hematopoietic progenitor cells (HPCs) differentiate into precursors [7], and then decreases during erythroblast maturation accompanied by cytoskeleton remodeling and loss of cytoplasmic-nuclear connections [7, 8]. Moreover, some studies have revealed that the self-renewal and differentiation of HSCs are affected by cell morphology and adhesion. Rho kinases controlling the cytoskeleton are required for the biological functions of HSCs and are particularly significant for enucleation during erythropoiesis [9–11]. Cell morphology and adhesion affect the polarity and proliferation of HSPCs [12–14]. Platelet factor 4 binds to HPCs, strengthens the adhesion of HPCs to the extracellular matrix, and ultimately modulates hematopoiesis [15]. However, whether the characteristics of a specific cell morphology and low adhesion are crucial factors in erythropoiesis and how they facilitate RBC development are still obscured.

Cell junction molecules are a type of cell adhesion molecules, and cell adhesion and cell junction systems dynamically and mutually interact [16]. Furthermore, tight junctions (TJs) play a role in recruiting various cytoskeleton and signaling molecules on their cytoplasmic surface and linking extracellular proteins with intracellular signaling pathways to the cytoskeleton [17]. Additionally, TJ members could affect the biological functions of HSCs. For example, intimate intercellular contact mediated by JAMs is required for efficient transduction of Notch signaling in HSCs, and deficiency of JAM1 results in impaired HSC specification [18, 19]. JAM2 regulates the maintenance of HSCs through heterotypic interactions with JAM3 [20, 21], and depletion of JAM3 leads to a sharp decrease in the frequency of myeloid progenitors in the bone marrow [22]. It is worth noting that the master hematopoietic regulator RUNX1 can respectively bind to TJP1, OCLN, and CLDN5 via the “TGGGGT” DNA sequence in the promoter region [23]. Overall, the overexpression of OCT4, the most important pluripotent transcription factor (TF), might alter the morphology and adhesion of hHFMSCs, potentially making the cells more prone to cytokine stimulation and differentiate towards the hematopoietic lineage.

In this study, we utilized next-generation sequencing combined with bioinformatics analysis, qPCR, and Western blotting, to investigate the interactions between cell adhesion, cytoskeleton, hematopoiesis, and pluripotency modulated by OCT4 in hHFMSCs. Ideally, elucidating the molecular mechanisms of OCT4-reprogrammed hHFMSCs differentiation into erythrocytes will not only help to understand the mechanism of RBC production, but also provide an experimental basis for hematopoiesis in vitro for future clinical applications.

## Materials and methods

### Cell isolation, characterization, and culture

hHFMSCs were isolated and characterized as previously described by our research group members [5, 24, 25]. Hairs with complete hair follicles were plucked off, and the root tissues were cut and intensively rinsed with phosphate-buffered saline (PBS, Sparkjade) containing 1% penicillin/streptomycin (Hyclone). Hair follicles were then transferred into 96-well plates and maintained in H-DMEM/F12 medium (Gibco) supplemented with 10% fetal bovine serum (FBS, Gibco), 100 U/mL penicillin-streptomycin (Sparkjade), and 10 ng/ml fibroblast growth factor-basic (bFGF, Acro Biosystem) at 37 °C with 5% CO_2_. Cells migrating from the dermal sheath or papilla were selected, pooled, and expanded. hHFMSCs were characterized with mesenchymal stem cell markers (CD29, CD44, CD73, CD90, CD105, CD166, etc.) and investigation of osteogenic, chondrogenic, and adipogenic differentiation. Transduced hHFMSCs were maintained in hHFMSCs medium on Matrigel (Corning)-coated culture plates, and floating transduced hHFMSCs were sorted from adherent ones during culture. The upper medium of adherent cells was collected and spun at 800 rpm for 5 min; the precipitation at the bottom of the tube was resuspended with hHFMSCS medium and seeded onto Matrigel-coated plates. When cells proliferated to 70–80%, they were digested with 0.25% trypsin-ethylene diamine tetra-acetic acid (EDTA) and subsequently subcultured. hHFMSCs were frozen with cryopreservation solution composed of 50% H-DMEM/F12 medium, 40% FBS and 10% dimethyl sulfoxide, and stored in liquid nitrogen at passages 0–2. Cells were thawed and expanded for experimentation at passages 2–3.

### Lentivirus production and transduction

Lentiviral vector pLV-EF1-OCT4-IRES-EGFP and packaging plasmids expressing gag-pol, pVSVG, and rev genes were transfected into 293 T cells, and the viral supernatants were harvested, filtered through a 0.45-μm filter and concentrated by ultracentrifugation. hHFMSCs seeded onto Matrigel-coated plates were infected with lentivirus expressing OCT4 in the presence of polybrene, and the EGFP-positive cells were observed using a fluorescence microscope. Passage 2 hHFMSCs were plated on Matrigel-coated plates and transfected with lentivirus. After 12 days, EGFP-positive cells were observed under a florescence microscope, and a subpopulation of floating and round or quasi-round cells emerged from hHFMSCS^OCT4^ after 14 days. hHFMSCs^OCT4^ were expanded for experiments at passages 7–8. Hereafter, hHFMSCs transduced with lentivirus encoding OCT4 are referred to as hHFMSCs^OCT4^, and floating transduced hHFMSCs and adherent transduced hHFMSCs are referred to as floating hHFMSCs^OCT4^ and adherent hHFMSCs^OCT4^ respectively.

### Immunofluorescence staining

For immunofluorescence staining, 10^4^ cells were washed three times in PBS and seeded onto slides. Slides were fixed with 4% paraformaldehyde for 10 min at room temperature, and subsequently permeabilized with 0.1% Triton X-100 (Sigma Aldrich) for 10 min at room temperature. After blocking with 1% bovine serum albumin, slides were incubated with ready-to-use primary antibody against OCT4 (Maxim) and PBS (as a negative control) at 4 °C overnight. The next day, Alexa Fluor 594-conjugated goat anti-mouse IgG (1:200 dilution, Abcam) was added onto slides for 1 h at room temperature to detect the primary antibody. Slides were then counterstained with DAPI to label the nuclei and imaged using a fluorescence microscope.

### Cell morphology observation

hHFMSCs in the well were monitored and photographed under an inverted microscope. After OCT4 transduction, floating hHFMSCs^OCT4^ and adherent hHFMSCs^OCT4^ were observed using an inverted microscope and a fluorescence microscope respectively.

### Flow cytometry analysis

A total of 10^7^ cells/mL were prepared for flow cytometry analysis. A 200-μL aliquot was added to 96-well plates per well, and then the light-scattering properties of the cells were measured by flow cytometry. In the flow cytometry assay, the value of forward scatter (FSC) is proportional to the size of the cells, so the FSC can be used to compare the relative size of the cells. FSC distribution histogram plots were generated by a computer with raw flow cytometry data. Results were performed from 3 individual experiments.

### Dissociation assay

Cell-cell adhesion was assayed through a dispase disassociate assay as previously described with minor modifications [26, 27]. 2 × 10^5^ cells of hHFMSCs, floating hHFMSCs^OCT4^, and adherent hHFMSCs^OCT4^ per well were seeded onto 24-well plates respectively and incubated over night until cells reached confluency. The next day, cells were treated with dispase II (2.4 U/mL, Sigma Aldrich) in an incubator and observed under an inverted microscope every 5 min, up to the time that floating hHFMSCs^OCT4^ disassociated into single cells; this took about 10 min. Following detachment from the plates, EDTA was added to interrupt the digestion; the cells were spun down at 1000 rpm for 5 min and resuspended with PBS. The number of single cells was counted using a hemocytometer. The percentage of single cells to the total number of cells was inversely proportional to cell-cell adhesion. Each sample was tested three times and three counts were taken per well.

### Cell adhesion assay

Cell-extracellular matrix adhesion was assayed as previously reported with minor modifications [28, 29]. 2 × 10^5^ cells per well were seeded onto Matrigel-coated 24-well plates and incubated for 6 h until hHFMSCs adhered to the plates and stable connection was formed. The plates were washed with digestive enzyme-free PBS for three times to remove unattached cells, the remaining cells were harvested, and the number was counted using a hemocytometer. The percentage of remaining cells to the total number of cells was proportional to cell-extracellular matrix adhesion. Each sample was tested three times and three counts were taken per well.

### RNA-seq and DEG analysis

hHFMSCs, floating hHFMSCs^OCT4^, and adherent hHFMSCs^OCT4^ were sent to Shanghai Oe-biotechnology (Shanghai, China) for RNA-seq analysis. Total RNA was extracted using the mirVana miRNA Isolation Kit and RNA integrity number (RIN) was evaluated by an Agilent 2100 Bioanalyzer. Samples with RIN greater than or equal to 7 were sequenced by an Illumina HiSeq X Ten sequencer (Illumina). The filtered clean reads were mapped to the reference genome database (accession number: GCF_000001405.38) by hisat2, v2.2.1.0 (http://ccb.jhu.edu/software/hisat2/index.shtml). Each transcript was normalized by FPKM to eliminate the influence of gene length and sequencing depth. The counts of each sample were mapped to the annotated genome after standardization and normalization. Finally, fold change (FC) and difference significance were used to screen the differentially expressed genes (DEGs). DEGs with FC value greater than 2 or lower than − 2, and a *P* value lower than 0.05 were considered significant. Each group of cells was sequenced with three independent biological replicates.

### GO and KEGG enrichment and network analysis

GO term and KEGG pathway enrichment analyses were carried out using the tool for Function Annotation in the DAVID (https://david.ncifcrf.gov/).The KEGG pathway maps were obtained from the KEGG database (http://www.kegg.jp/). Significant genes were visualized by the STRING database (http://string-db.org/), and a network was constructed using Cytoscape software (https://cytoscape.org/).

### Expression validation using qPCR

Total RNA was extracted from 5 × 10^6^ cells treated with 1 mL TRIzol (Sparkjade, Shandong, China), and the purity and concentration were determined by a NanoDrop 2000 (Thermo Fisher Scientific). cDNA was synthesized with the PrimeScript RT reagent Kit (+ gDNA Eraser) and then subjected to qPCR using TB Green® Premix Ex Taq™ II (Takara). The gene mRNA levels were determined using 50 ng of cDNA on an Applied Biosystems 7300. All template amplifications were conducted in triplicate with a three-step PCR process, which included one cycle of 95 °C for 30 s, 40 cycles of 95 °C for 5 s and 60 °C for 31 s, and one final cycle of 95 °C for 15 s, 60 °C for 1 min, and 95 °C for 15 s. Using GAPDH expression as a normalization control, the relative expression was calculated as 2^−∆∆Ct^. The primer sequences are provided in Table 1.

**Table 1:** qPCR primer sequences

Gene	Forward primer	Reverse primer
TJP1	CAACATACAGTGACGCTTCACA	CACTATTGACGTTTCCCCACTC
TJP2	TTTCCCGACACCCCAAAGTAG	CACCCTGCTTCCTTGAGACC
TJP3	AGCTACAAGCCTCGGGTTCC	GCTGTTCCCAGATGGTCAGC
JAM1	ATGGGGACAAAGGCGCAAG	CAATGCCAGGGAGCACAACA
JAM3	TCCAGCAATCGAACCCCAG	CTTGTCTGCGAATCCGTAATGAT
CLDN5	CTCTGCTGGTTCGCCAACAT	CAGCTCGTACTTCTGCGACA
CLDN6	TGTTCGGCTTGCTGGTCTAC	CGGGGATTAGCGTCAGGAC
CLDN7	AGTTAGGAGCCTTGATGCCG	GCACAGGGAGTAGGATACGC
CLDN11	CATTGCCAGTTGACTGCCTG	ATCCGGATGCAGGGAAGAAC
ACTN2	GACATCGTGAACACCCCTAAAC	CCGCAAAAGCGTGGTAGAA
E-cadherin	ACCACGGGCTTGGATTTTGA	GGAGGTGGTGAGAGAGACCT
ROCK1	AAGTGAGGTTAGGGCGAAATG	AAGGTAGTTGATTGCCAACGAA
RUNX1	GTGGGTACGAAGGAAATGACTCAAA	GCAGCGTGGTAAAAGAAATCATTGAG
GAPDH	CCATGTTCGTCATGGGTGTGA	CATGGACTGTGGTCATGAGT

### Western blot analysis

Total proteins were isolated with Radio Immunoprecipitation Assay buffer (Solarbio) supplemented with 1% phenylmethylsulfonyl fluoride (Solarbio), followed by centrifugation to remove cell debris. Then, protein extracts were subjected to protein estimation using a BCA protein assay kit (Solarbio). Sodium dodecyl sulfate-polyacrylamide gel electrophoresis (SDS-PAGE) was carried with equal amounts of proteins, and then proteins were transferred to a polyvinylidene fluoride membrane (Millipore). Upon blocking with 5% nonfat milk, the membrane was incubated with primary antibody overnight at 4 °C. After washing three times with TBST, the membrane was incubated with HRP-conjugated secondary antibody at room temperature for 2 h. Finally, the blots were developed with Immobilon Western Chemilum HRP Substrate (Millipore). Differential protein expression was calculated as a ratio normalized to β-actin protein expression. The optimal dilutions of antibodies used for Western blot analysis are provided in Table 2.

**Table 2:** Optimal dilutions of antibodies used for Western blot analysis

Antibodies	Dilution rates
Rabbit Anti-Mouse TJP1 antibody (Cell Signaling Technology)	1:2500
Rabbit Anti-Mouse CLDN5 antibody (Abcam)	1:3000
Rabbit Anti-Mouse CLDN11 antibody (Abcam)	1:3000
Rabbit Anti-Mouse JAM1 antibody (Abcam)	1:5000
Rabbit Anti-Mouse RUNX1 antibody (Abcam)	1:1000
Rabbit Anti-Mouse β-actin antibody (EpiZyme)	1:3000
Goat Anti-rabbit IgG antibody (Bioss)	1:3000

### Statistical analysis

All numerical data from flow cytometry, disassociation assay, cell adhesion assay, qPCR, and Western blot are presented as means ± standard deviations. Comparisons between the two groups were performed with independent sample *t* tests. Pearson correlation analysis was used to produce the correlation coefficient between two different samples, and a hypothesis test was performed on whether there is a difference in the expression level of each gene in two different samples through negative binomial distribution. GraphPad Prism 8.0.2 was used to generate histograms. *P* values inferior to 0.05 were considered significant.

## Results

### Overexpression of OCT4 causes changes in morphology and adhesion in hHFMSCs

**Fig. 1:**
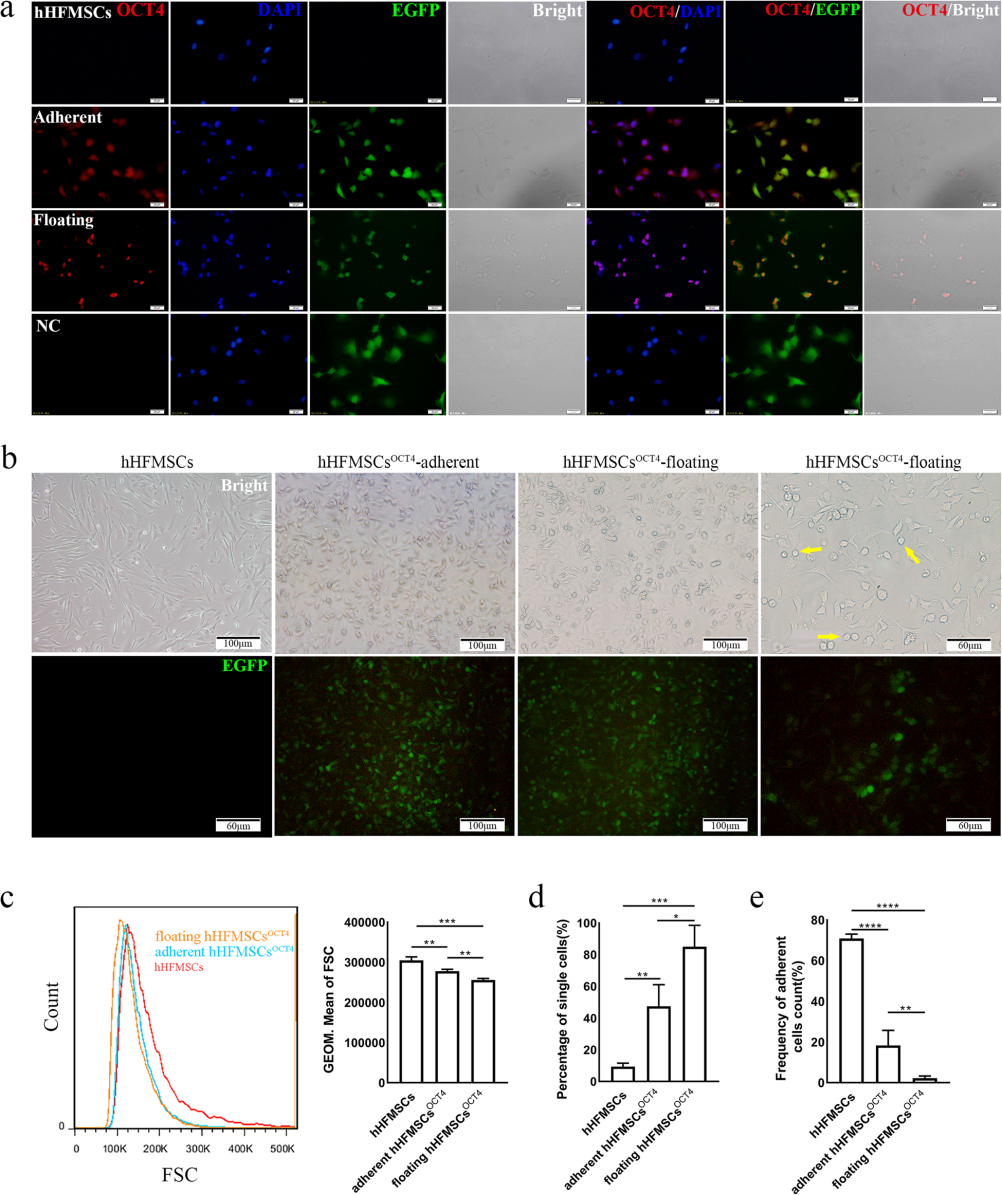
Cell morphology and adhesion alterations. **a** Immunofluorescence of OCT4 expression and localization in hHFMSCs, adherent hHFMSCs^OCT4^, and floating hHFMSCs^OCT4^. Adherent, adherent hHFMSCs^OCT4^; Floating, floating hHFMSCs^OCT4^; NC, negative control. Red represents OCT4, blue for DAPI and green for EGFP. **b** The morphology of hHFMSCs was gradually altered after the transduction of OCT4. Yellow arrows indicate round floating hHFMSCs^OCT4^. The first four upper pictures were taken to observe the morphology of the cells under an optical microscope, and the last lower four pictures were taken to observe the expression of EGFP after OCT4 transduction under a fluorescence microscope. These pictures were all taken from randomly selected fields. **c** Flow cytometric plot of three groups of hHFMSCs and geometric mean of the FSC. **d** Percentage of single cells adhered to the plates after treatment with dispase. The percentage of disassociated individual cells to the total number of cells is negatively correlated with cell-to-cell adhesion. e Percentage of cells adhered to the Matrigel-coated plates after rinsing with PBS three times. *P < 0.05, **P < 0.01, ***P < 0.001, ****P < 0.0001. The error bars represent the standard deviations of measurements in three separate sample runs (*n* = 3)

EGFP-positive signals indicated that OCT4 had been transduced into hHFMSCs. Immunofluorescence staining showed that both floating hHFMSCs^OCT4^ and adherent hHFMSCs^OCT4^ expressed OCT4, while the expression of OCT4 was not detected in hHFMSCs (Fig. 1a). And the protein OCT4 was located in the nuclei of the cells. Cellular morphologic changes were monitored using an optical microscope. The spindle-shaped cells became polygonal after transduction, and a population of small floating round or quasi-round cells emerged from the adherent polygonal cells (Fig. 1a, b). We then determined the relative size of these cells by flow cytometry. The data showed that the size of adherent hHFMSCs^OCT4^ was notably smaller than that of hHFMSCs, while floating hHFMSCs^OCT4^ were smaller than adherent cells (Fig. 1c), which was consistent with what we observed under an optical microscope. At the same time, cell adhesion changed as a portion of the cells gradually became suspended in the medium, so dissociation and adhesion assays were carried out to detect cell adhesion. The percentage of single cells was higher in adherent hHFMSCs^OCT4^ (47.5%) than in hHFMSCs (9.4%), and the percentage was higher in floating hHFMSCs^OCT4^ (85%) than in adherent hHFMSCs^OCT4^ (Fig. 1d). In the adhesion assay, the percentage of remaining cells was lower in adherent cells (18.4%) than in hHFMSCs (70.9%), while it was lower in floating hHFMSCs^OCT4^ (2.3%) than in adherent hHFMSCs^OCT4^ (Fig. 1e). The above results validated that the morphology of OCT4-reprogrammed hHFMSCs changed and adhesion decreased. It is worth noting that it was the population of floating hHFMSCs^OCT4^ with low-adhesion prone to transdifferentiate towards erythroid lineage after stepwise stimulation by cocktails of hematopoietic cytokines. Accordingly, stretched cell morphology and strong adhesion might be the negative factors affecting erythropoiesis.

### Transcripts in cells with diverse morphology and adhesion are quite different

**Fig. 2:**
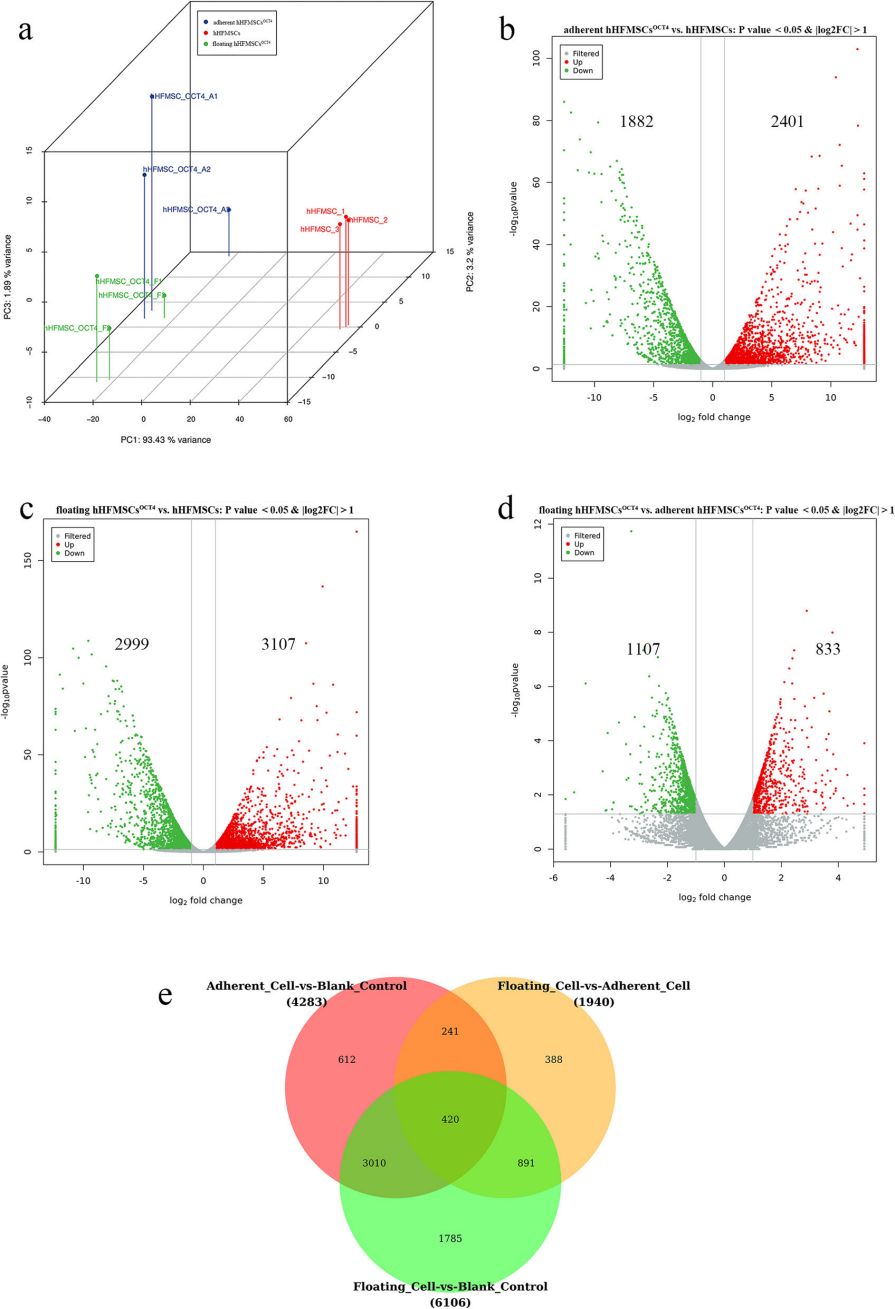
Comparisons of the transcripts and DEG analysis. **a** Principal component of samples distributed in three dimensions; *n* = 3. **b**–**d** Differential expression of genes in the three different comparison groups represented by volcano plots; *n* = 3. Colors are representative by *P* < 0.05 and |FC|>2. **e** Venn diagram is used to represent common and specific DEGs between the different comparison groups; *n* = 3

To investigate the role of cell morphology and adhesion during erythrocyte differentiation from hHFMSCs^OCT4^, RNA-seq was performed and the DEGs were sorted. First, to compare the similarity of samples within a group and the diversity of each group, the principle components were analyzed. As shown in the three-dimensional distribution (Fig. 2a), the closer distances of cells within each group implied better repetitiveness, and the distances between every two groups were significantly greater, especially when adherent hHFMSCs^OCT4^ and floating hHFMSCs^OCT4^ were compared with hHFMSCs, respectively. Based on these data combined with the correlation coefficient and clustering of correlation provided in Additional file 1, OCT4 induced hHFMSCs to derive novo cell populations; remarkably, cells with different morphology and adhesion might possess distinct gene transcripts.

Next, DEGs were sorted among the three groups. When we compared adherent hHFMSCs^OCT4^ with hHFMSCs, 2401 upregulated genes and 1882 downregulated genes were identified (Fig. 2b), and 3107 upregulated genes and 2999 downregulated genes were determined in floating hHFMSCs^OCT4^ compared to hHFMSCs (Fig. 2c), indicating that OCT4 conferred considerable changes of transcriptome in the whole genome to hHFMSCs. Importantly, 833 upregulated genes and 1107 downregulated genes were also identified when floating hHFMSCs^OCT4^ were compared with adherent hHFMSCs^OCT4^ (Fig. 2d). Although the number is smaller, it would definitely play a considerable role in floating cells. Venn diagram analysis revealed a total of 612 and 388 group-specific DEGs for adherent hHFMSCs^OCT4^ vs. hHFMSCs and floating hHFMSCs^OCT4^ vs. adherent hHFMSCs^OCT4^, respectively (Fig. 2e). In particular, 1785 group-specific DEGs were identified in floating hHFMSCs^OCT4^ vs. hHFMSCs, which was a much larger number than that in the other two comparison groups. This considerable number of DEGs probably brings about significant changes in biological function to OCT4-reprogrammed hHFMSCs. Especially, the group-specific DEGs may yield tremendous changes to floating hHFMSCs^OCT4^ when compared with hHFMSCs. In the DEGs analysis, it is likely that the common and group-specific DEGs collectively affected the morphological characteristics and subsequent transdifferentiation.

### Floating hHFMSCs^OCT4^ and adherent hHFMSCs^OCT4^ acquire different pluripotency and differentiation tendency

**Fig. 3:**
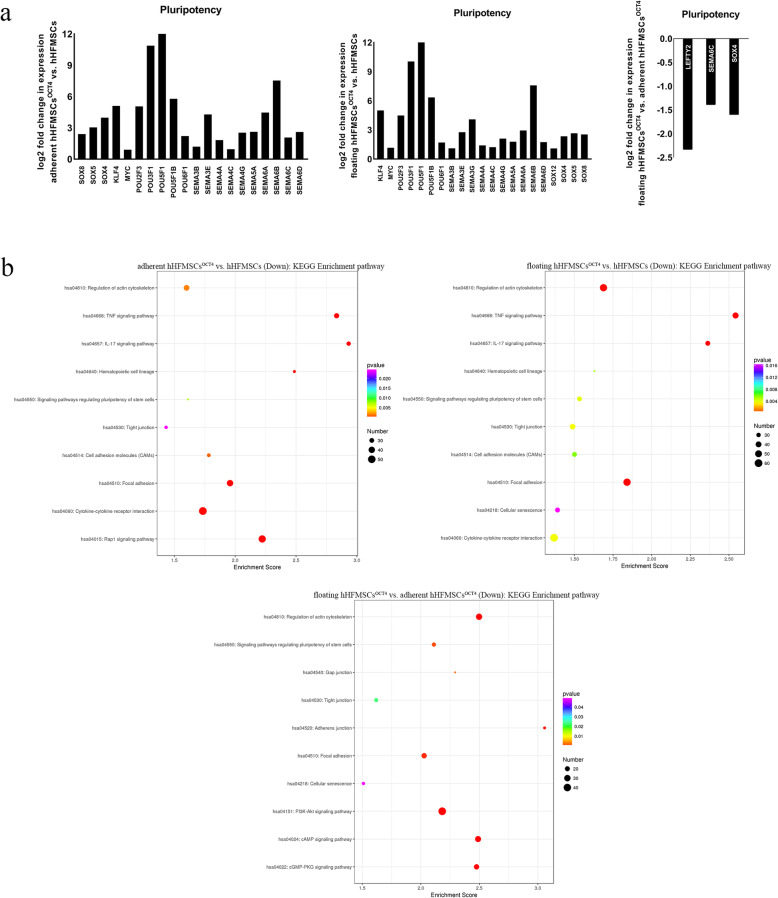
Expression of pluripotent genes and KEGG enrichment analysis. **a** Pluripotency-related gene expression in hHFMSCs, adherent hHFMSCs^OCT4^, and floating hHFMSCs^OCT4^. **b** Bubble chart representing the significantly enriched pathways from the KEGG analysis. The color of dots in the bubble chart indicates the significance of the enriched category, and the size represents the scale of enriched genes in the terms

Cells with different transcripts might possess different pluripotency after transduction of the key pluripotent transcription factor OCT4. Consequently, we focused our analysis on the expression of related genes in cells with different morphology and adhesion. hHFMSCs expressed negligible levels of OCT4, LEFTY2, SOX18, POU3F2, and SEMA4D (Additional file 2: Table S1), and both adherent hHFMSCs^OCT4^ and floating hHFMSCs^OCT4^ expressed higher levels of pluripotent genes, including LEFTY2, KLF4, MYC, POUs, SEMAs, and SOXs, than hHFMSCs (Fig. 3a). Some of the pluripotent genes, such as LEFTY2, SOX4, and SEMA6C, however, were downregulated in floating hHFMSCs^OCT4^ compared with adherent hHFMSCs^OCT4^ (Fig. 3a), which was validated by KEGG enrichment analysis as downregulated genes were enriched in the term signaling pathways regulating pluripotency of stem cells in floating hHFMSCs^OCT4^ vs. adherent hHFMSCs^OCT4^ (Fig. 3b). These results suggested that OCT4-reprogramed hHFMSCs acquired pluripotency, but lost some of it after adherent cells transformed into the floating subset. The DEGs were then clustered according to their GO terms using DAVID, and the top 10 GO terms related to differentiation and development enriched with upregulated genes and downregulated genes were separately analyzed. Upregulated DEGs were involved in three germ layers differentiation terms (retinal rod cell development, trachea gland development, negative development of endothelial cell differentiation morphogenesis, cartilage development involved in endothelia development, etc.) in hHFMSCs^OCT4^ compared with hHFMSCs (Table 3 and Additional file 2: Table S2, S3). This enrichment result verified again the pluripotency of hHFMSCs^OCT4^, and the upregulated genes in floating cells were specially enriched in the terms T-helper 1 cell differentiation and regulation of erythrocyte differentiation (Table 3), implying a potential for erythropoietic differentiation.

**Table 3:** Differentiation- and development-related GO terms enriched of upregulated DEGs in floating hHFMSCs^OCT4^ versus hHFMSCs

id	Term	Count	Genes	*P* value	Enrichment score
GO:0046548	Retinal rod cell development	3	NAGLU, TRPM1, RORB	0	6.2558
GO:0045063	T-helper 1 cell differentiation	3	LEF1, SEMA4A, IL18R1	0	6.2558
GO:0060351	Cartilage development involved in endochondral bone morphogenesis	3	SHOX2, TRPV4, COL2A1	0	6.2558
GO:1903225	Negative regulation of endodermal cell differentiation	2	COL5A1, COL5A2	0	6.2558
GO:0060538	Skeletal muscle organ development	2	PAX3, CNTFR	0	6.2558
GO:0061153	Trachea gland development	2	EDA, LEF1	0	6.2558
GO:1902871	Positive regulation of amacrine cell differentiation	2	DLX1, DLX2	0	6.2558
GO:0045646	Regulation of erythrocyte differentiation	2	LYN, P4HTM	0	6.2558
GO:0003431	Growth plate cartilage chondrocyte development	2	POC1A, COL27A1	0	6.2558
GO:0045605	Negative regulation of epidermal cell differentiation	2	EZH2, DLL1	0	6.2558

### Gradual downregulation of the tight junction pathway in adherent hHFMSCs^OCT4^ and floating hHFMSCs^OCT4^

**Fig. 4:**
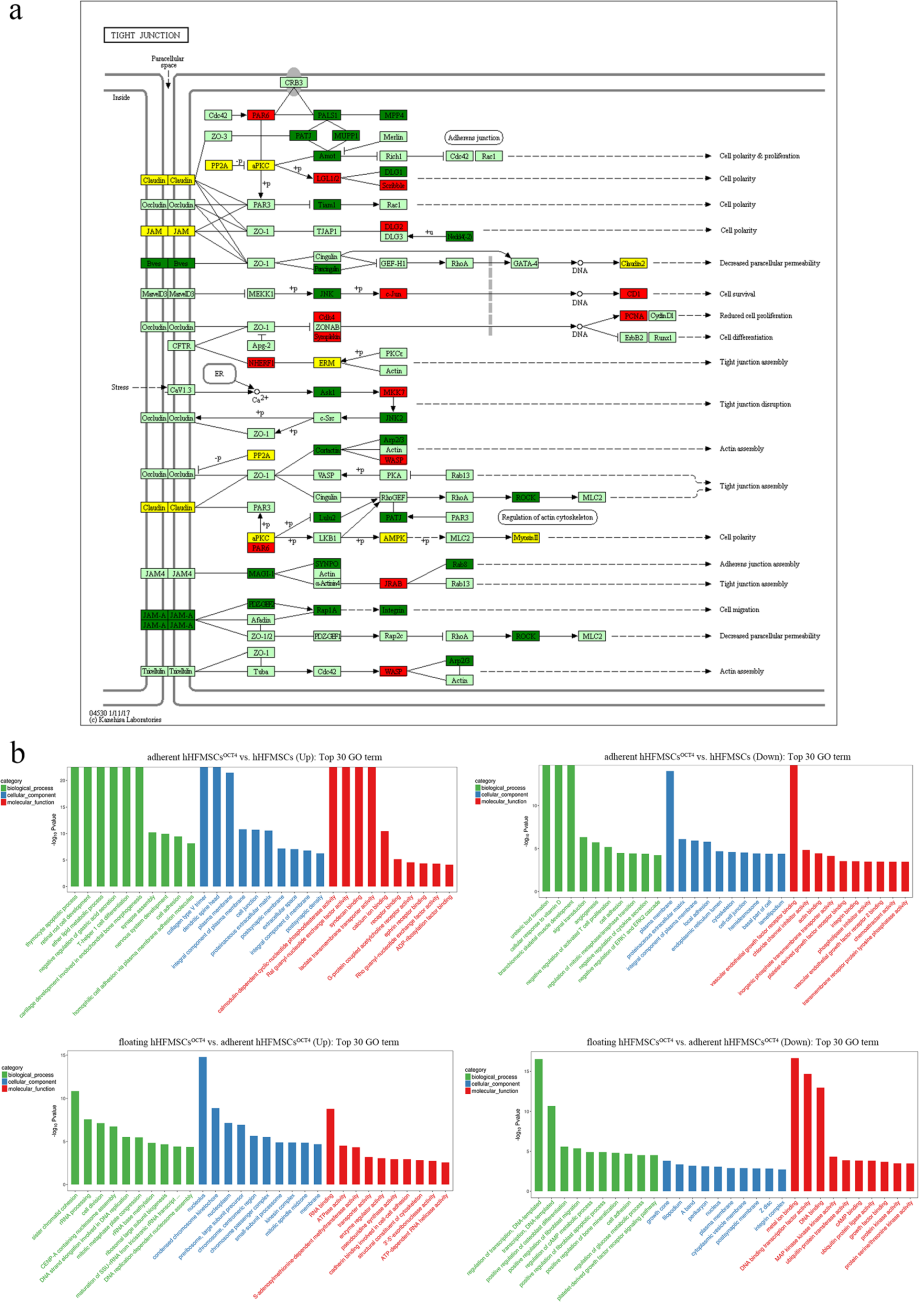
GO analysis of DEGs and TJ pathway annotation. **a** Top 10 enriched GO terms covering biological process, cellular component, and molecule function in adherent hHFMSCs^OCT4^ vs. hHFMSCs, floating hHFMSCs^OCT4^ vs. hHFMSCs, and floating hHFMSCs^OCT4^ vs. adherent hHFMSCs^OCT4^ for upregulated genes and downregulated genes, respectively. **b** Annotated TJ pathway according to the KEGG database. Red boxes indicate upregulated genes, green boxes indicate downregulated genes, and yellow boxes indicate both upregulated and downregulated genes and paralogs

It is worth noting that the TJ pathway was found to be downregulated by KEGG analysis in these three comparison groups (Fig. 3b). The TJ pathway was annotated through the KEGG database and the DEGs were annotated (Fig. 4a); there were 12 upregulated genes and 20 downregulated genes in the TJ pathway. The results clearly showed that TJ genes were dynamically expressed, and several programs, such as cell proliferation, adhesion, cytoskeleton, cell polarity, paracellular permeability, and most importantly cell differentiation, were involved.

Tight junctions, generally known for their fence function controlling cellular matter diffusion, can also modulate cell adhesion and the cytoskeleton [17, 30]. KEGG analysis also revealed that downregulated genes were enriched in cell signaling pathways including regulation of actin cytoskeleton, cell adhesion molecules, and focal adhesion in adherent hHFMSCs^OCT4^ vs. hHFMSCs, as well as pathways of regulation of actin cytoskeleton, gap junction, adherens junction, and focal adhesion in floating hHFMSCs^OCT4^ vs. adherent hHFMSCs^OCT4^ (Fig. 3b).

To further explore the role of cell morphology and adhesion during erythropoiesis in hHFMSCs^OCT4^, the top 10 GO terms, covering biological process, molecule function, and cellular component, are displayed in Fig. 4b. Downregulated genes were obviously enriched in relevant terms, such as cell adhesion, focal adhesion, cytoskeleton, and cell-cell junction in adherent hHFMSCs^OCT4^ compared to hHFMSCs. In addition, downregulated genes were significantly enriched in the term cell adhesion in floating hHFMSCs^OCT4^ relative to adherent hHFMSCs^OCT4^, suggesting the sharp decrease in adhesion of floating hHFMSCs^OCT4^. These results verified the changes in cell morphology and adhesion of hHFMSCs^OCT4^, which were consistent with our observations. Therefore, the morphology- and adhesion-related genes aroused corresponding alterations in OCT4-reprogrammed hHFMSCs and facilitated the switch between adherent and floating subpopulations.

### The expression validation of the TJ pathway and cytoskeleton genes as well as adhesion molecules

**Fig. 5:**
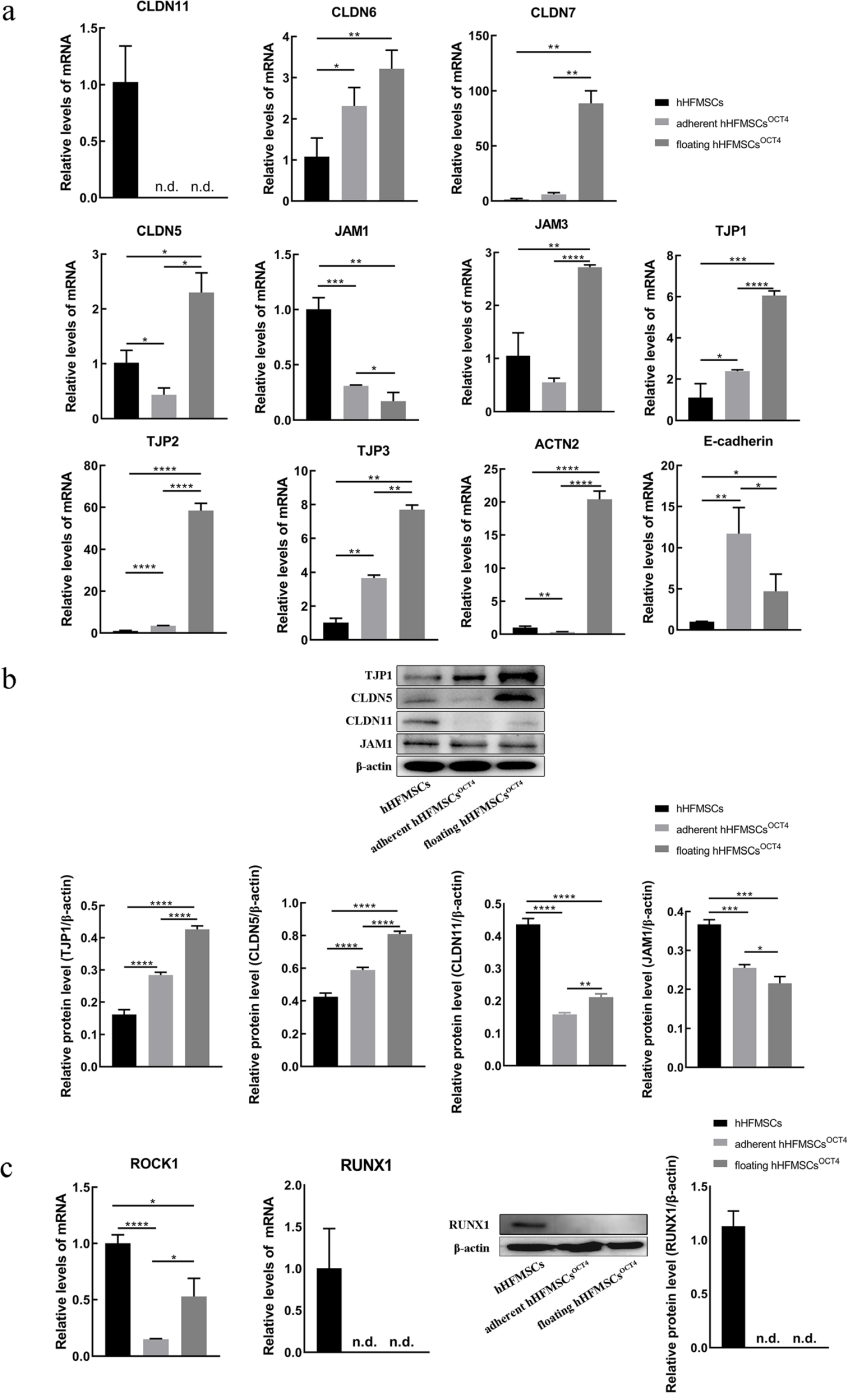
Gene expression validation by qPCR and Western blot. **a**, **b** Histograms are mapped to validate the expression of the DEGs by qPCR and Western blot, respectively. **c** Expression validation of hematopoietic factors. *P < 0.05, **P < 0.01, ***P < 0.001, ****P < 0.0001. n.d., undetected. The error bars represent the standard deviations of measurements in three separate sample runs (*n* = 3)

Cell junction molecules including TJ members are involved in cell adhesion and could directly affect cell adhesion [16]. Therefore, we performed qPCR to detect the mRNA expression levels of selected genes associated with TJs (CLDNs, JAMs and TJPs), adhesion, or cytoskeleton (Fig. 5a). Compared with hHFMSCs, the expression of TJ member CLDN11 was significantly decreased in floating hHFMSCs^OCT4^ and adherent hHFMSCs^OCT4^, while both CLDN6 and CLDN7 were downregulated. Especially, CLDN5 was downregulated in adherent hHFMSCs^OCT4^ and then upregulated in floating hHFMSCs^OCT4^. The expression levels of JAM1 and JAM3, which play an important role in the commitment of lineage specification and cellular signaling transduction in HSCs, were respectively decreased and increased in floating hHFMSCs^OCT4^ relative to hHFMSCs. Moreover, the expression levels of TJP1, TJP2, and TJP3, core members associated with the cytoskeleton and intracellular signaling transduction, were remarkably upregulated 5.4-fold, 59.4-fold, and 7.6-fold in floating hHFMSCs^OCT4^.

Next, Western blotting was carried out to further validate the expression of the selected proteins in hHFMSCs after OCT4 transduction. As shown in Fig. 5b, both TJP1 and CLDN5 were upregulated in hHFMSC^OCT4^. The TJP1 protein was upregulated 1.75-fold and 2.6-fold in adherent hHFMSCs^OCT4^ and floating hHFMSCs^OCT4^, respectively, and the CLDN5 protein was upregulated 1.38-fold and 1.9-fold. However, CLDN11 and JAM1 were downregulated in hHFMSCs^OCT4^. The CLDN11 protein was downregulated 2.76-fold and 2.1-fold in adherent hHFMSCs^OCT4^ and floating hHFMSCs^OCT4^, respectively, and the JAM1 protein was downregulated 1.4-fold and 1.7-fold. When floating hHFMSCs^OCT4^ were compared with adherent hHFMSCs^OCT4^, there were also some differences in expressions of TJ members. The expressions of TJP1 and CLDN5 continued to increase and CLDN11 showed subtle increase, while JAM1 were downregulated. These results indicated disrupted molecular homeostasis of the TJ pathway upon OCT4 transduction.

We also found that the cytoskeleton gene ACTN2 increased more than 20-fold in floating cells, and the expression of E-cadherin increased in adherent hHFMSCs^OCT4^ and then decreased in floating hHFMSCs^OCT4^ (Fig. 5a), implying that the changes in adhesion and cytoskeleton of these cells.

### Hematopoietic factor expression in floating hHFMSCs^OCT4^ and adherent hHFMSCs^OCT4^

To reveal the influence of the TJ pathway on hematopoietic differentiation, we detected the expression levels of the terminal erythroid differentiation-related gene ROCK1 and the essential hematopoietic development gene RUNX1, both of which are in the downstream of the TJ pathway (Fig. 4a). Expression of these two genes were found decreased in floating cells (Fig. 5c), indicating that the state of hematopoietic program was not yet triggered, although OCT4 conferred pluripotency in this group of cells to some degree as other pluripotency genes MYC, KLF4, etc., were upregulated as shown in previous sequencing data. We also detected the expression of RUNX1 protein (Fig. 5c), and the expression tendency was consistent with the mRNA level.

### A putative regulatory network in floating hHFMSCs^OCT4^

**Fig. 6:**
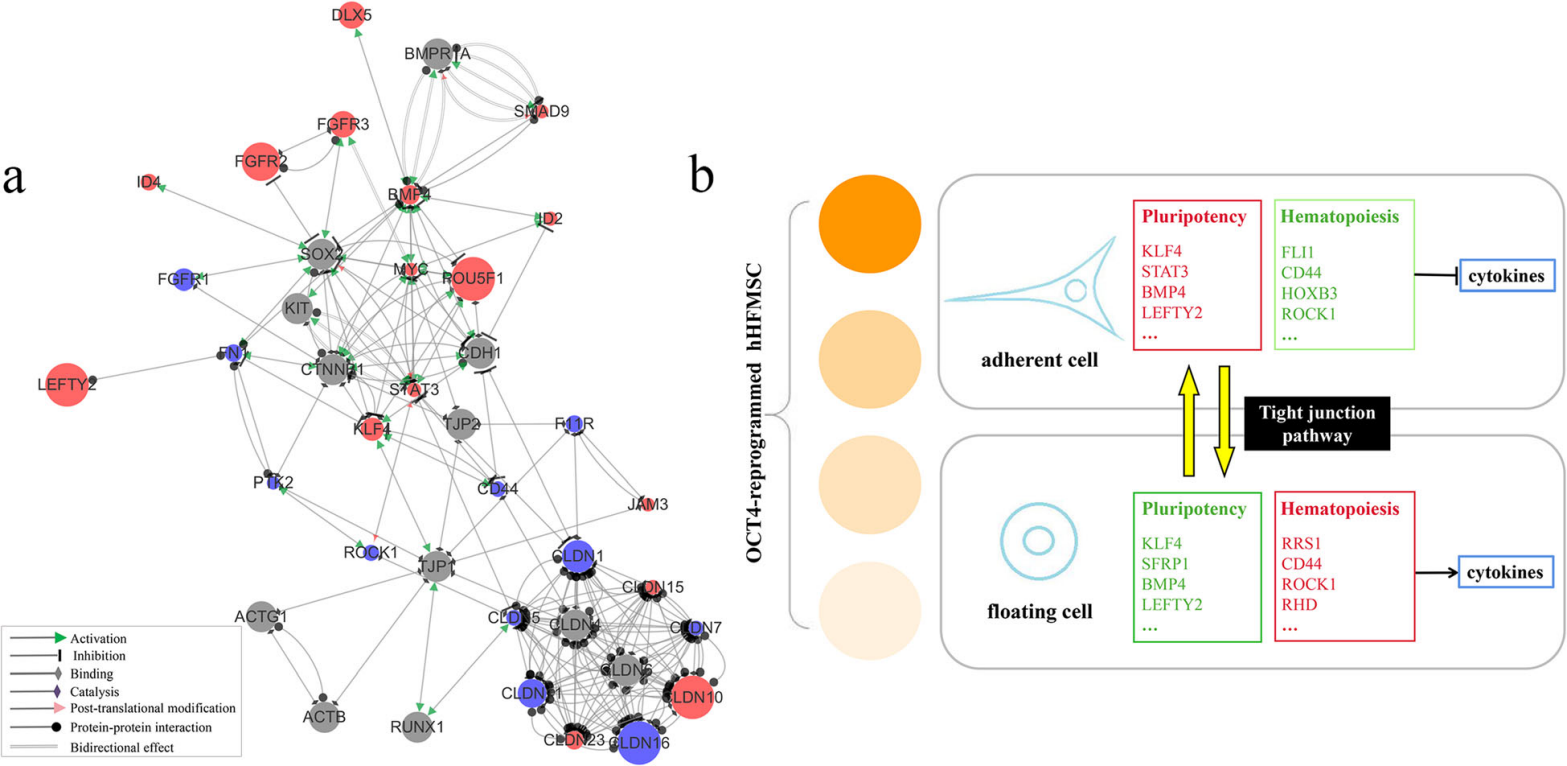
Network analysis and hypothetical model construction. **a** A pluripotency regulatory network of floating hHFMSCs^OCT4^. Red dots indicate genes that are upregulated, blue dots are downregulated genes, and undifferentially expressed genes are shown in gray. The size of the dot is proportional to the absolute value of the log2-fold change value. **b** Hypothetical model in which OCT4-reprogrammed hHFMSCs modulate pluripotency and hematopoietic capacity by regulating morphology and adhesion via the TJ pathway. The red box represents genes upregulated genes in cells, and green one represents downregulated genes. Circles with different shades of color represent changes in cell adhesion, and light to dark represent low to high

Although pluripotency-, cell morphology-, and cell adhesion-related genes have been identified, the internal correlations between these factors are still unknown. Therefore, we visualized the significant DEGs in floating hHFMSCs^OCT4^ vs. hHFMSCs and constructed a network (Fig. 6a). Firstly, there are interactions within the TJ pathway (CLDNs/TJPs/JAMs). Secondly, the TJ pathway members can interact with adhesion-, cytoskeleton-related molecules, such as PTK2, CDH1, CTNNB1, ACTB, and ACTG1. Thirdly, tight junction proteins (TJP1 and TJP2) and cell adhesion genes (CDH1 and FN1) as well as cytoskeleton genes (CTNNB1, ACTB, and ACTG1) could interact with pluripotency genes and regulate pluripotency-related transcription factors OCT4, SOX2, MYC, and KLF4. Finally, cell junction and adhesion molecules can bind to or interact with hematopoietic-related factors (such as CD44, CD117, RUNX1, and ROCK1). It shows that OCT4 may indirectly regulate the expression of TJP1 through the target gene KLF4, and the TJ pathway with TJP1 as the core can interact with cell junction-, adhesion-, and cytoskeleton-related molecules, thereby affecting cell morphology and adhesion. And TJP1 and CLDN5 could interact with KLF4 and STAT3, respectively, and then influence the expression of other pluripotent genes, indicating a bridge role of TJ pathway between cell morphology, adhesion, and pluripotency. On the other hand, TJP1 and CLDN5 could regulate the expression of hematopoietic factors RUNX1 and ROCK1, respectively, thus linking cell morphology and adhesion to hematopoietic differentiation. This regulatory network elucidates the molecular mechanisms of mutual conversion between adherent hHFMSCs^OCT4^ and floating hHFMSCs^OCT4^ and the balance maintenance between pluripotency and hematopoietic differentiation centered to OCT4/TJP1.

In summary, transduction of OCT4 brought about great differences in morphology and adhesion to hHFMSCs, whereby two subsets of cells appeared and gained pluripotency, with floating cells losing some of pluripotency and acquiring hematopoietic tendency to some extent. The dynamically expressed TJ pathway before erythropoietic inducement might act as a pivotal point of changes in cell morphology and adhesion, resulting in damaged pluripotency in floating cells and probable constructed hematopoietic capacity in a TJP1-dependent way.

## Discussion

It was reported that floating hHFMSCs^OCT4^ treated with hematopoietic cytokines could directly transdifferentiate into enucleated RBCs expressing β-globin [6]. Here, we investigated the correlation between cell morphology, adhesion, pluripotency, and hematopoiesis to elucidate the mechanisms of erythropoiesis in OCT4-reprogrammed hHFMSCs. The results verified the alterations in morphology and adhesion in hHFMSCs^OCT4^, and the corresponding changes in gene expression were detected by RNA-seq, qPCR, and Western blot. In-depth analysis of sequencing data showed that the expression of genes related to pluripotency changed in hHFMSCs^OCT4^ with diverse adhesion and morphology, and floating cells displayed hematopoietic differentiation potential. All these results suggest the possible effects of changes in cell adhesion, junctions, and cytoskeleton on pluripotency and hematopoietic differentiation in OCT4-reprogrammed hHFMSCs.

Pluripotency-related DEGs dynamically expressed in two subpopulations of hHFMSCs^OCT4^, and pluripotency was presumably reduced in floating cells compared with adherent cells. In humans, direct conversions of mature somatic cells to another type of cells using the single factor OCT4, a key pluripotency TF, have been widely developed [31, 32]. Although not shown to be involved in physiological hematopoiesis, OCT4 is capable of promoting the expression of essential hematopoietic regulators in supportive culture conditions [31]. Transduction of the individual factor OCT4 allows hHFMSCs to transdifferentiate into RBCs under multiple hematopoietic induction conditions, providing an optional way to generate RBCs in vitro for transfusion [6]. Evolutionary conservation analysis was performed to identify a relative subset of targets for OCT4 and other POU proteins that link the regulation of cell-cell adhesion to differentiation [33]. Whether OCT4 regulates pluripotency through adhesion or regulates both in parallel is difficult to determine, however, analysis of the conserved OCT4 network indicates that the regulation of differentiation and adhesion is inseparable [33]. This may partially explain the relatively decreased pluripotency in floating hHFMSCs^OCT4^.

Attenuated pluripotency prompts cells to be more likely to differentiate into a certain type of cell. Blood cells are well known to grow in suspension and erythropoietic precursors show a progressive reduction in cell and nuclear size during erythropoiesis [34], extremely similar to the sequencing results and our observations upon floating hHFMSCs^OCT4^. A number of molecules associated with cell adhesion and the cytoskeleton in hematopoietic cells are proved essential for erythropoiesis under homeostasis and stress [35–39]. Embryonic hematopoiesis involves activation of a hematopoietic transcriptional program, followed by major morphological changes and breakage of the tight junctions [40]. Tight junction members, TJPs, CLDNs, and JAMs, control self-renewal, proliferation, and recovery from stress in hematopoietic cells, including ESCs, HSCs, and pluripotent stem cells [22, 23, 41–45]. Our results of sequencing analysis, qPCR, and Western blot clearly demonstrate dynamically expressed TJ members in hHFMSCs^OCT4^ with divergent morphology and cell adhesion. Mutual conversion between adherent hHFMSCs^OCT4^ and floating hHFMSCs^OCT4^ seems to account for the dynamic TJ pathway, causing disparate morphology and adhesion, and ultimately regulating the expression of genes related to pluripotency and hematopoiesis (Fig. 6b). However, it is still unclear whether these morphological alterations initiate the process of erythropoiesis or are the inevitable phenomenon accompanying erythroid hematopoiesis since erythropoiesis requires membrane biogenesis, establishment of cell polarity, and cytoskeleton assembly during differentiation and enucleation [46–48]. More research should be conducted to address this problem.

In mammals, RUNX1 is identified as a core regulator of hematopoiesis that is essential for the initiation of the hematopoietic program in embryonic hematopoiesis [49–51], and RUNX1 could promote lymphoid development in HSCs while suppressing myeloid development [50]. Additionally, there is bidirectional negative regulation between TJP1 and RUNX1 [23, 52]. Therefore, upregulated TJP1 suppressed the expression of RUNX1 in floating cells, implying the ascendancy for myeloid differentiation and there may be other hematopoietic initiation mechanisms in floating cells. And downregulation of terminal erythroid differentiation-related gene ROCK1 probably suggests that follow-up hematopoietic cytokines are required for floating cells to differentiate into erythrocytes. Consequently, the morphological changes potentially confer a certain degree of pluripotency to floating hHFMSCs^OCT4^, accompanied by probable enhanced sensitivity to hematopoietic cytokines and thus trigger the hematopoietic program.

Herein we present a hypothetical model (Fig. 6b), in which the TJ pathway might regulate hematopoiesis in OCT4-reprogrammed hHFMSCs by transforming cell morphology from adherent polygonal or fusiform to floating small and round. Although further experiments should be followed up, such as knock-out of TJP1, investigations of pluripotency and hematopoietic capacity, study of morphological changes effect on intercellular signal transduction and detection of sensitivity to hematopoietic cytokines.

## Conclusions

We have developed potential mechanisms of erythropoiesis in OCT4-reprogrammed hHFMSCs, where changes in cell morphology and adhesion were involved through the TJ pathway. This study characterized possible hematopoietic molecular mechanisms in hHFMSCs^OCT4^ in vitro, providing comprehensive insight into the potential role of the TJ pathway during erythropoiesis.

## Supplementary information

**Additional file 1: Fig. S1.** Comparisons of the transcripts. (a) Correlation coefficient between samples displayed in a heatmap. The closer the correlation coefficient is to 1, the higher the sample similarity is. (b) Hierarchical clustering of correlation of the three groups of cells. The closer the sample clustering distance is, the higher the sample similarity is.

**Additional file 2: Table S1.** Pluripotency related genes expression in hHFMSCs, adherent hHFMSCs^OCT4^ and floating hHFMSCs^OCT4^. **Table S2.** Differentiation and development relative GO terms enriched of upregulated DEGs in adherent hHFMSCs^OCT4^ versus hHFMSCs. **Table S3.** Differentiation and development relative GO terms enriched of upregulated DEGs in floating hHFMSCs^OCT4^ versus adherent hHFMSCs^OCT4^.

## Data Availability

The datasets supporting the conclusions of this article are available in the SRA database, with unique accession code PRJNA615033 and hyperlink to dataset(s) in https://dataview.ncbi.nlm.nih.gov/object/PRJNA615033?reviewer=oru002jv1ibpksdhnj42usqa1o. All other data are concluded in this article.

## Abbreviations

RBCs: Red blood cells
HSPCs: Hematopoietic stem and progenitor cells
MSCs: Mesenchymal stem cells
ESCs: Embryonic stem cells
HSCs: Hematopoietic stem cell
hHFMSCs: Human hair follicle mesenchymal stem cells
hHFMSCs^OCT4^: HPCs: hematopoietic progenitor cells
TJs: Tight junctions
TF: Transcription factor
bFGF: Fibroblast growth factor-basic
PBS: Phosphate-buffered saline
FBS: Fetal bovine serum
EDTA: Ethylene diamine tetra-acetic acid
FSC: Forward scatter
RIN: RNA integrity number
FC: Fold change
DEGs: Differentially expressed genes
KEGG: Kyoto Encyclopedia of Genes and Genomes
GO: Gene Ontology
