# Correction to: RNA-seq reveals tight junction-relevant erythropoietic fate induced by OCT4 in human hair follicle mesenchymal stem cells

**DOI:** 10.1186/s13287-020-02079-7

**Published:** 2021-01-15

**Authors:** Xiaozhen Yu, Pengpeng Sun, Xingang Huang, Hua Chen, Weiqing Huang, Yingchun Ruan, Weina Jiang, Xiaohua Tan, Zhijing Liu

**Affiliations:** 1 Department of Pathology, Qingdao Municipal Hospital affiliated with Qingdao University, 1 Jiaozhou Road, Qingdao, 266000 Shandong China; 2 Department of Critical Care Medicine, Qingdao Center Medical Group, affiliated with Qingdao University, 127 Siliunan Road, Qingdao, 266042 Shandong China; 3 Department of Pathology, College of Basic Medical Sciences, Qingdao University, 308 Ningxia Road, Qingdao, 266000 Shandong China

**Correction to: Stem Cell Res Ther (2020) 11:454**

**https://doi.org/10.1186/s13287-020-01976-1**

The original article [1] contains incorrect affiliation information; the correct affiliations can be viewed in this article.
