# Supplementary figures and images for: RNA-seq reveals tight junction-relevant erythropoietic fate induced by OCT4 in human hair follicle mesenchymal stem cells
Source: Stem Cell Res Ther. 2020 Oct 27;11:454. doi: 10.1186/s13287-020-01976-1 (PMC7590701; doi:10.1186/s13287-020-01976-1)

a

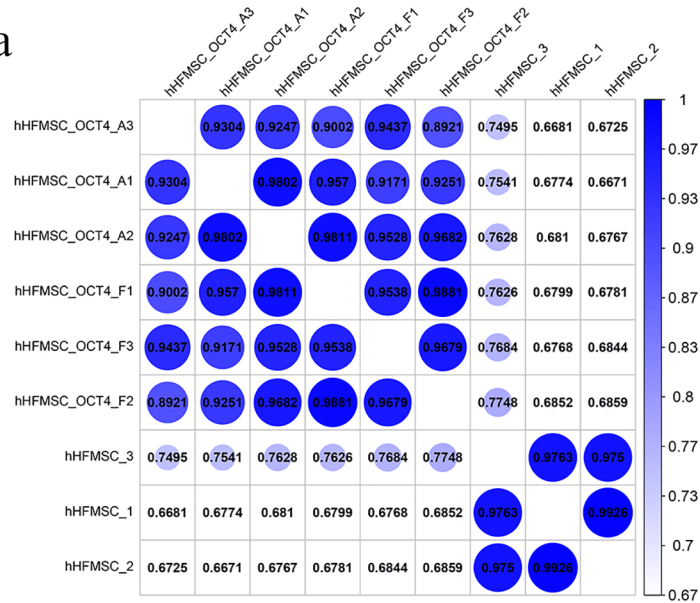

b

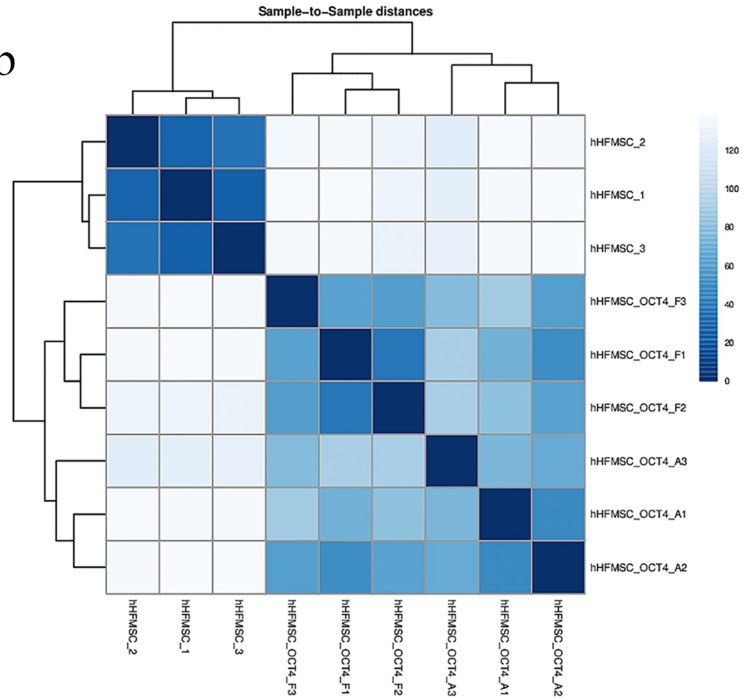

Supplement: Supplementary file 1 — Additional file 1: Fig. S1. Comparisons of the transcripts. (a) Correlation coefficient between samples displayed in a heatmap. The closer the correlation coefficient is to 1, the higher the sample similarity is. (b) Hierarchical clustering of correlation of the three groups of cells. The closer the sample clustering distance is, the higher the sample similarity is. [file 13287_2020_1976_MOESM1_ESM.pdf]
