# Supplementary material for: RNA-seq reveals tight junction-relevant erythropoietic fate induced by OCT4 in human hair follicle mesenchymal stem cells
Source: Stem Cell Res Ther. 2020 Oct 27;11:454. doi: 10.1186/s13287-020-01976-1 (PMC7590701; doi:10.1186/s13287-020-01976-1)
Supplement: Supplementary file 2 — Additional file 2: Table S1. Pluripotency related genes expression in hHFMSCs, adherent hHFMSCsOCT4 and floating hHFMSCsOCT4. Table S2. Differentiation and development relative GO terms enriched of upregulated DEGs in adherent hHFMSCsOCT4 versus hHFMSCs. Table S3. Differentiation and development relative GO terms enriched of upregulated DEGs in floating hHFMSCsOCT4 versus adherent hHFMSCsOCT4. [file 13287_2020_1976_MOESM2_ESM.pdf]

Table S1. Pluripotency related genes expression in hHFMSCs, adherent hHFMSCs^{OCT4} and floating hHFMSCs^{OCT4}.

Cell type	LEFTY2	SOX18	SEMA4D	POU3F2	POU5F1
hHFMSC_1	0	0	0	0	0.073
hHFMSC_2	0	0	0	0	0.020
hHFMSC_3	0	0	0	0	0.087
hHFMSC^{OCT4}-adherent_1	23.1801	2.573	0.142	0.132	305.831
hHFMSC^{OCT4}-adherent_2	21.978	3.509	0.094	0.062	375.245
hHFMSC^{OCT4}-adherent_3	15.004	0.879	0.155	0.021	224.463
hHFMSC^{OCT4}-floating_1	2.389	2.644	0.224	0.166	504.263
hHFMSC^{OCT4}-floating_2	1.659	1.758	0.089	0.180	469.818
hHFMSC^{OCT4}-floating_3	8.693	1.849	0.112	0.178	367.820

Table S2. Differentiation and development relative GO terms enriched of upregulated DEGs in adherent hHFMSCs^{OCT4} versus hHFMSCs.

id	Term	Count	Genes	*P*-value	Enrichment score
GO:0046548	retinal rod cell development	3	NAGLU, TRPM1, RORB	0	8.1412
GO:0060351	cartilage development involved in endochondral bone morphogenesis	3	SHOX2, TRPV4, COL2A1	0	8.1412
GO:0061005	cell differentiation involved in kidney development	2	GLI3, GLIS2	0	8.1412
GO:0060538	skeletal muscle organ development	2	PAX3, CNTFR	0	8.1412
GO:0061153	trachea gland development	2	EDA, LEF1	0	8.1412
GO:0071599	otic vesicle development	2	AHI1, COL2A1	0	8.1412
GO:0061309	cardiac neural crest cell development involved in outflow tract morphogenesis	2	HES1, HAND2	0	8.1412
GO:0060873	anterior semicircular canal development	1	GLI3	0	8.1412
GO:0060875	lateral semicircular canal development	1	GLI3	0	8.1412
GO:1903010	regulation of bone development	1	GLI3	0	8.1412

P-value is a measurement of enrichment of the GO analysis.

Table S3. Differentiation and development relative GO terms enriched of upregulated DEGs in floating hHFMSCs^{OCT4} versus adherent hHFMSCs^{OCT4}.

id	Term	Count	Genes	*P*-value	Enrichment score
GO:0021541	ammon gyrus development	1	LRP8	0	23.8456
GO:2000287	positive regulation of myotome development	1	DMRT2	0	23.8456
GO:2000355	negative regulation of ovarian follicle	1	HYAL3	0	23.8456

	development				
GO:1903011	negative regulation of bone development	1	RGN	0	23.8456
GO:0072076	nephrogenic mesenchyme development	1	FOXD1	0	23.8456
GO:0072213	metanephric capsule development	1	FOXD1	0	23.8456
GO:0043009	chordate embryonic development	1	CENPU	0	23.8456
GO:0043045	DNA methylation involved in embryo development	2	ZFP57, TRIM28	0.0024	11.9228
GO:0001890	placenta development	4	CCNF, PHLDA2, ASCL2, LHX4	0.0083	4.7691
GO:0003431	growth plate cartilage chondrocyte development	1	POC1A	0.0083	11.9228

P-value is a measurement of enrichment of the GO analysis.
